# Daily Versus Alternate Day Thyroxine Therapy to Maintain Euthyroidism in Children With Congenital Hypothyroidism

**DOI:** 10.5812/ijem.9499

**Published:** 2013-10-01

**Authors:** Devi Dayal, Lokesh Saini, Savita Verma Attri, Baljinder Singh, Anil Kumar Bhalla

**Affiliations:** 1 Departments of Pediatrics, Postgraduate Institute of Medical Education and Research, Chandigarh, India; 2 Departments of Nuclear Medicine, Postgraduate Institute of Medical Education and Research, Chandigarh, India

**Keywords:** Hypothyroidism, Thyroxine, Children

## Abstract

**Background:**

Daily administration of thyroxine has proven efficacy in treatment of children with hypothyroidism. However, the possibility of treatment with longer dosing intervals that offers flexibility and choice in maintaining euthyroid state has not been tested in children.

**Objectives:**

To study the efficacy of an alternate day regimen to maintain euthyroidism in children with congenital hypothyroidism

**Patients and Methods:**

Forty patients given alternate day therapy, while 30 children continued on their daily regimen were followed up at monthly intervals for 3 months. Clinical and laboratory assessments were performed at each follow up visit.

**Results:**

The clinical and anthropometric parameters remained similar in both groups of patients during the study indicating a maintained euthyroid state clinically. The thyroid profiles also remained within normal limits suggesting biochemical euthyroidism status with alternate day therapy. However the baseline serum aminotransferase levels showed mild elevation in patients on alternate day regimen and the difference persisted during the follow up visits. Higher HDL and lower TC and LDL levels suggested some beneficial effect of alternate day schedule on lipid profiles.

**Conclusions:**

In short-term, alternate day schedule can be effectively used to maintain clinical and biochemical euthyroid state in children with congenital hypothyroidism beyond 4 years of age.

## 1. Background

Traditionally, thyroxine administration is done as a once-daily dose. With this regimen, if started early, normal growth and intellectual development is expected (1, 2). Given the proven effectiveness of daily-dose regimen, this has been continued to be followed over last several decades. The basis of this daily administration of thyroxine has however remained unclear since the elimination half life of thyroxine is about 7 days and its biological effects may last even longer (3, 4). In addition, there is a strong evidence of auto regulation of peripheral conversion of thyroxine (T4) to triiodothyronine (T3) with increasing conversion rates at low serum T4 levels and decreasing conversion when T4 is elevated (5). Also, large doses of thyroxine are usually well tolerated (6-8). Together these properties suggest the possibility of using dosing intervals longer than the traditional 24 hours. But while it is possible to maintain near euthyroidism at the tissue level using even weekly dosing of thyroxine, the possibility of toxicity between 2-6 days of administration cannot be excluded completely (7). In an observation on an infant, biochemical hyperthyroidism soon after the dose and hypothyroidism at the end of each week of weekly therapy was postulated for the poor developmental outcome (9). To circumvent aforementioned problems of a weekly regimen, a twice weekly dosing schedule has been suggested (10). The studies on longer dosing regimens in hypothyroidism have only been conducted in adult patients (6-8). In fact weekly or biweekly regimens may not be ethically justifiable in children due to concerns about growth and development. Alternate day dosing schedule may be plausible because practitioners generally recommend giving doubled dose of thyroxine next day in case of a missed dose. 

Daily life-long administration may be quite burdensome for some families and can lead to non-adherence to therapy (11). In a recent observation on hypothyroid patients, non-compliance was suggested to be the most common cause of lack of adequate response to thyroxine replacement therapy (12). Longer dosing intervals may improve compliance and could be particularly advantageous to parents or caregivers because thyroxine has to be administered by them as children are generally unable to dose themselves.

## 2. Objectives

We therefore planned this study to test the efficacy of an alternate day regimen and explore the possibility of using this regimen for maintaining euthyroidism in children with congenital hypothyroidism.

## 3. Patients and Methods

This prospective case control study was carried out from July 2010 to June 2011. Seventy patients suffering from congenital hypothyroidism and on follow up in the Pediatric Endocrinology Clinic of our hospital were enrolled. Inclusion criteria were age more than 4 years and clinically and biochemically euthyroid status for at least 6 months. Those on additional drugs known to interfere with thyroxine metabolism e.g. antiepileptics and the ones unable to come for regular follow up visits were excluded. The study was approved by the Institute’s Ethics Committee. 

Patients in one group (Group A) were given thyroxine on alternate days; the dose of thyroxine was double of their previous daily dose. The controls (Group B) continued to take thyroxine once a day. Adjustments in thyroxine dose in both groups were done as per previous routine to keep total T4 in upper half of normal range and TSH at lower normal range,usually less than 2 µIU/mL. All children were followed up at monthly intervals. At each follow up visit, a thorough clinical assessment was performed specially targeted at symptoms and signs suggestive of hypo or hyperthyroid state as per a prestructuredproforma.

Laboratory evaluation consisted of estimation of total T3, total T4, thyroid stimulating hormone (TSH), aspartate aminotransferase (AST), alanine aminotransferase (ALT), total cholesterol (TC), high-density lipoprotein (HDL), low-density lipoprotein (LDL), very low-density lipoprotein (VLDL) and triglycerides (TG) levels at each monthly follow up visit.

### 3.1. Statistical Analysis

The continuous data was first checked for its normalcy by Kolmogorov Smirnov test. For normally distributed data, the comparison between study and control group was made by Student’s t-test. For Skewed data, the distribution of the two groups was compared using Mann-Whitney test. The results of symptoms questionnaire were analyzed either by Chi-Square or Fisher test, whichever applicable. McNemar test was applied for significance of changes from visit to visit. Repeated Measure ANOVA was applied to find the trend of the measurable data over visits and to compare the two groups. A ‘P’ value of < 0.05 was considered significant in all the tests. The statistical analysis was performed using Statistical Package for Social Sciences (SPSS Inc., Chicago, IL version 15 for windows).

## 4. Results

Group A consisted of 40 patients and Group B had 30 patients. None of the patients dropped out after enrollment. The baseline characteristics of the study population are depicted in Table 1. The mean weight of the children in 2 groups remained similar at 1 ^st ^(24.67+11.48 kg versus 25.82+13.56 kg, P = 0.704), 2nd (25.18+11.68kg versus 26.01+13.00 kg, P = 0.780) and 3rd (25.33+11.70 kg versus 26.36+12.86 kg, P = 0.728) follow up visits. Similarly there was no significant difference in the heights of children in the 2 groups at all time points in the study. None of the children in either group showed any alterations in clinical characteristics (sleep, appetite, sweating, activity, stooling pattern and cold or heat preferences) over the study period. 

**Table 1.** Baseline Characteristics of the Study Population

Parameter	Group A	Group B	P value
**Age, mo, Mean (SD)**	91.10±34.36	97.80±36.72	0.44
**Gender, No. (%)**			0.47
Male	14 (35%)	13 (43.3%)	
Female	26 (65%)	17 (56.7%)	
**Weight, kg, Mean (SD)**	25.06±11.61	26.06±13.14	0.737
**Height, cm, Mean (SD)**	119.1±18.01	120.50±19.23	0.750
**Levothyroxine dose, μg/kg/d, Mean (SD)**			
At start	3.02±0.86	3.03±0.86	0.96
At end	3.00±0.82	3.07±0.91	0.73

### 4.1. Thyroid Profiles 

The mean T3, T4 and TSH levels did not differ significantly in the 2 groups at baseline as well as at all the 3 follow up visits (Table 2). 

**Table 2.** Comparison of Parameters of Thyroid Profiles between Two Study Groups at Baseline andduring Follow Up Visits

Parameter	Time Point	Group A (Mean ± SD)	Group B (Mean ± SD)	P value
**T3, nmol/L**	Baseline	1.46±0.29	1.49± 0.35	0.753
	FU visit 1	1.44±0.36	1.55±0.39	0.252
	FU visit 2	1.50±0.31	1.50±0.54	0.994
	FU visit 3	1.44±0.35	1.41± 0.38	0.722
**T4, µg/dL**	Baseline	10.01±1.37	10.51± 0.77	0.058
	FU visit 1	9.96±1.95	10.98±1.20	0.091
	FU visit 2	9.84±2.01	10.32±0.78	0.172
	FU visit 3	10.23±1.20	10.23± 1.20	0.996
**TSH, mIU/mL**	Baseline	2.73± 1.28	3.13± 1.07	0.159
	FU visit 1	2.89±1.86	2.88±1.69	0.976
	FU visit 2	2.53±1.25	3.03±1.33	0.117
	FU visit 3	2.76±1.63	3.48± 1.51	0.061

### 4.2. Aspartate and Alanine Transaminase Levels

The baseline AST and ALT levels in Group A were higher, compared to Group B and the difference persisted during the follow up visits (Table 3). 

### 4.3. Lipid Profiles

At baseline, HDL levels were higher and TC and LDL levels were lower in Group A as compared to Group B and this difference persisted (more significantly for HDL) during the 3 follow ups (Table 4). Other parameters of lipid profile (VLDL and TG) remained similar in the 2 groups at all time points (Table 4). 

**Table 3.** Comparison of AST and ALT Levels between Two Study Groups at Baseline andduring Follow Up Visits

Parameter	Timepoint	Group A, Mean ±SD	Group B, Mean ±SD	P value
**AST, IU/L**	Baseline	41.78± 8.27	34.4± 8.85	0.001
	FU visit 1	43.98± 11.41	36.40± 10.42	0.005
	FU visit 2	41.23± 8.96	34.30± 11.08	0.007
	FU visit 3	40.15±9.74	32.50± 8.10	0.001
**ALT, IU/L**	Baseline	36.68± 6.61	27.31± 7.89	0.0001
	FU visit 1	38.13±7.56	28.13±9.49	0.0001
	FU visit 2	35.83±9.66	27.33±8.50	0.0003
	FU visit 3	36.10±7.14	26.47± 7.59	0.0001

**Table 4.** Comparison of Various Parameters of Lipid Profiles Between Two Study Groups at Baseline and During Follow Up Visits

Parameter	Timepoint	Group A, Mean ±SD	Group B, Mean ±SD	P value
**HDL, mg/dL**	Baseline	46.95± 6.14	39.74± 5.39	0.000
	FU visit 1	45.33±9.23	38.43±6.69	0.001
	FU visit 2	46.98±6.63	40.00±5.03	0.000
	FU visit 3	48.55±4.20	40.80± 5.93	0.000
**LDL, mg/dL**	Baseline	75.24±12.06	81.97±7.39	0.005
	FU visit 1	74.93±18.04	84.38± 9.12	0.006
	FU visit 2	74.90±14.18	80.30±11.01	0.078
	FU visit 3	75.91±10.72	81.24± 7.02	0.014
**VLDL, mg/dL**	Baseline	20.5±6.31	19.31± 3.43	0.316
	FU visit 1	22.72±8.44	19.97±3.68	0.071
	FU visit 2	20.02±6.35	19.38±3.55	0.598
	FU visit 3	18.78±6.21	18.58± 3.68	0.869
**TG, mg/dL**	Baseline	101.33± 32.31	96.41± 17.34	0.417
	FU visit 1	109.28±41.86	99.87±18.39	0.210
	FU visit 2	100.77±31.30	96.90±17.85	0.516
	FU visit 3	93.94±30.99	92.48± 19.10	0.810
**TC, mg/dL**	Baseline	144.64± 18.36	156.28± 16.22	0.007
	FU visit 1	146.68±25.90	158.20±17.81	0.031
	FU visit 2	142.23±17.94	155.97±17.82	0.002
	FU visit 3	145.03±17.47	154.70± 16.16	0.020

## 5. Discussion

The clinical and anthropometric parameters remained similar in both groups of patients during the study indicating maintained euthyroid state clinically. The thyroid function tests also continued to be within acceptable limits at all 3 follow up visits in both study arms, suggesting biochemical euthyroidism status with alternate day therapy. Our observations are similar to some earlier studies in older patients with hypothyroidism (6, 7). However due to financial constraints we could not estimate free T3 and free T4 levels which were measured by the previous investigators.

To study the acute effects of thyroid hormones at the tissue level, we measured several markers that have been used as validated parameters previously (8). These markers of tissue effects (AST, ALT, TC, HDL, LDL, VLDL and TG) have been shown to be discriminatory even in subclinical hypothyroid states (13-16). In the present study, AST and ALT levels were found to be higher in Group A as compared to Group B at all 3 time points in follow-ups, indicating an inadequate control of hypothyroid state at the tissue level. It is however noteworthy that even the first estimation of aminotransferases levels at inclusion into the study showed a similar difference in two groups. It is therefore difficult to attribute significance to the difference in levels that occurred during the study. The mean age of children in group A was slightly lower as compared to those in group B and may partly explain higher levels of serum aminotransferases in group A. Serum levels are generally higher in younger children and decrease with increasing age (17). The higher levels of aminotransferases in younger children are usually considered benign (17). In our patients on alternate day regimen, the higher serum aminotransferase levels may also be attributed to a less well controlled hypothyroid state as compared to those on daily dose regimen. Similar observations of slightly increased liver enzymes and normal thyroid function tests have been made in earlier studies on adult patients which may not have much clinical significance (18).

Both quantitative and qualitative lipid alterations occur in overt and subclinical hypothyroidism and contribute to cardiometabolic risk in adult patients (19). However, normalization of TSH with levothyroxine treatment has not been consistently associated with a beneficial effect on serum lipid levels in subclinically hypothyroid patients (20). In this context, the higher HDL and lower TC and LDL levels in children who received alternate day regimen suggested marginally beneficial effects on the lipidogram in the study population.

Since this was a short term study we did not measure ALP and osteocalcin levels which are more reflective of chronic effects of hypothyroidism at tissue level (20). The age group of more than 4 years was chosen to avoid any effect of change in therapeutic regimen on the growing brain as most of the thyroid hormone-dependent brain development is complete by 3 years of age (21, 22).

In conclusion, these observations suggest that alternate day schedule can be effectively used to maintain short term clinical and biochemical euthyroid state in children with congenital hypothyroidism beyond early childhood. This regimen may have some beneficial effects on lipid profiles and negligible effects at tissue level.
